# Variations and value improvement in back pain care in one area of England

**DOI:** 10.1007/s43999-024-00038-w

**Published:** 2024-02-27

**Authors:** Jane Ferreira, Tim Wilson, Erica Ison, David Oates, Sarah Kirkman, Gwyn Bevan

**Affiliations:** 1 NHS Sherwood Forest NHS Foundation Trust, King’s Mill Hospital, Mansfield Road, Sutton in Ashfield, Nottinghamshire, NG17 4JL England; 2 NHS Arden-GEM System Support Service, Leicester, England; 3 Oxford Value and Stewardship Programme, Oxford, England; 4 NHS North of England Commissioning Support Unit, Durham, England; 5 London School of Economics, London, England

**Keywords:** Musculoskeletal care, Back pain, Value improvement, QALY, Unwarranted variation, Equity

## Abstract

The pursuit of value and equity have been put on a legal footing in the NHS with the arrival of the legal duty for all in the NHS to improve health and well-being of the population served, to provide fair access to high quality healthcare, and to use resources sustainably and efficiently. Recognising this we used analysis of variation to help us understand the degree to which we were fulfilling our new duty for people with back pain in Mid-Nottinghamshire and where there might be opportunities for value improvement.

MSK Together is a group of clinical and managerial representatives from providers, purchasers, local government, and patients who work collectively to optimise the use of resources for people with MSK conditions in Mid-Nottinghamshire. Back pain is the third largest burden of disease in the locality, and the largest cause of disability, so it is of strategic importance to MSK Together—we wanted to know about, and act on, opportunities for value improvement across the population of people with back pain.

In 2019/20, after adjusting for age and sex, we found a greater than three-fold variation among general practices in age-sex standardised rates of all hospital service usage for back pain conditions. When looking at a four-year period (2016/17–2019/20), the observed variation increased to eight-fold for (with narrow 95% confidence intervals). When looking at procedures (e.g., surgery or injections), the standardised variation among general practices was six-fold in 2019/20. The deprivation score of the general practice (a heterogenous measure given the mixed neighbourhoods many general practices serve) showed little correlation to the rates observed and did not appear to justify the variation.

When we looked at the deprivation of the neighbourhood from which the individuals receiving back pain procedures came, there appeared to be a weak correlation in terms of lower rates of intervention in the least-deprived compared with the most-deprived communities. This correlation was not tested statistically. People receiving hospital services for back pain appeared to receive the first episode of care most often in their 40s (working age), compared with people from the least-deprived areas who received care most commonly in their 60s (approaching retirement).

When we looked at the interventions provided in Mid-Nottinghamshire for back pain, 29 interventions were provided to 17,225 people. Using a recent NICE evaluation of cost-effectiveness of back pain interventions, we established that, of these 29 interventions, 16 have evidence of improving the quality of life, for nine there was no evidence of benefit or harm, for three there was evidence that they do not provide an improvement in quality of life, and for one there was possible evidence of harm. The total cost of interventions was estimated at £4.5 million and, using the evidence from the NICE review, the total quality adjusted life year (QALY) gain to the treated population of people with back pain was calculated to be 4,571 QALYs.

After discussions among the MSK Together group, it was agreed that some interventions could be stopped or scaled down, and new interventions introduced (in particular, in more-deprived neighbourhoods). Within the same estimated cost envelope of £4.5 million, the QALY gain was predicted to increase to 7702 QALYs and, by targeting QALY-related interventions to people from deprived neighbourhoods, reduce inequity (and therefore health inequalities).

Using variation helped us identify areas for improvement and generated a momentum for change among the MSK Together group. By examining what we were doing, the associated costs, and the likely QALY benefits (from research evidence), we identified lower value interventions to stop or reduce and new interventions to introduce, achieving greater health gain for people with back pain with no additional resource requirements.

## Introduction

In the English NHS, there is now a legal duty to consider, in decision making, three aims:

1. The impact on the health and well-being of the populations served, including inequalities in those outcomes;
2. The impact on access to high-quality healthcare services, including inequalities in access; and
3. Using NHS resources sustainably and efficiently [1].

To realise these aims, we developed a population approach that focused on the needs of the whole population with back pain. We sought efficiency by improving outcomes that matter to the people served with the resources available (value) [2]. In Nottinghamshire and England, back pain is the third highest specific condition contributing to the burden of disease (after ischaemic heart disease and COPD) [3]. From a population of 340,233 in 2012, 62,343 (18.3%) people reported back pain—a very common condition [4]. Addressing inequity in treatment of back pain is an opportunity for improving health inequalities from treating unmet needs in more deprived groups [5, 6].

Our group (MSK Together) was created from clinical and managerial representatives from all the providers and purchasers involved in MSK care in the Mid-Nottinghamshire area. The aim was to create a more integrated, higher quality, service. We recognised that we might also be able to increase the value of services provided for people with MSK conditions, including back pain, through optimising the use of resources.

Our hypothesis was that by understanding variation, we would be able to identify issues to address and build the case for change. We examined variations in treatment across general practices and the level of deprivation in the neighbourhoods of people with back pain. For different interventions, we examined the population and use of resources to inform ways of reallocating resources to increase the value within the current total use of resources. This would then enable us to redirect resources from lower, or low, value interventions, to higher value interventions to increase value.

## Method

A list of interventions for back pain conditions, and their associated codes, was developed for hospital out-patient and in-patient episodes using treatment speciality codes, OPCS, HRG subchapter and ICD-10 codes (see Table 1). The search was limited to adults (18 years and over) and excluded emergency activity.

**Table 1:** Table of treatment specialities, and OPCS, HRG and ICD-10 codes used in the analysis

Treatment Specialties included: (110) Trauma & Orthopaedics (Trauma is excluded as per the scope of the project) (191) Pain Management (410) Rheumatology *NB. Where "multiple specialties exist for a single spell", this is the treatment specialty at Discharge*
Admission Method codes (Elective = 11-12-13)
HRG Sub Chapters AB - Pain Management HB - Spinal Procedures and Disorders (Historic Code pre 17/18) HC - Spinal Procedures and Disorders HD - Musculoskeletal and Rheumatological Disorders HE - Orthopaedic Disorders HN - Orthopaedic Non-Trauma Procedures Excludes: Patients with CANCER Diagnoses-in any episode position ICD Diagnoses codes (C00-C97), or (D37-48) are flagged as cancer and excluded for spell they relate to
Outpatient activity is restricted to cover GP Referrals only (National Source of referral code 3) and it excludes Fracture clinics activity—these are identified by Local Specialty codes on the Medway PAS system (Local specialty 9F) which is national agreement (110FRA)
ICD 10 codes for pain: Back pain: M40 (all postural) M421-4219 (osteochondrosis) M43-436, M471-4799 (spondylosis with or without myelopathy or radiculopathy) M480-4809 (stenosis), M50 (discs with or without radiculopathy, myelopathy) M51, M53, M541, M542, M543, M544, M545, M546, M548, M549 Fibromyalgia: M797

The English Hospital Episode Statistics (HES) database was searched using these codes for people registered with the two purchasing groups in Mid-Nottinghamshire (NHS Newark and Sherwood and NHS Mansfield and Ashfield, covering 40 general practices) over the time-period 1 April 2015 to 31 March 2020 [7]. The deprivation status of the area of residency of the person was was identified using Lower layer Super Output Areas (LSOAs). LSOAs are geographic areas, comprising between 400 and 1,200 households and have a usually resident population between 1,000 and 3,000 persons (the average is around 1600). Each LSOA is allocated and IMD score and decile [8]. Additionally, we used the deprivation score of the general practice with which the patient was registered to determine whether deprivation affected clinical practice. Because general practices often provide services to adjacent, but markedly different, neighbourhoods, the general practice deprivation score is a poor marker of deprivation of individual patients, but a reasonable marker of the impact of deprivation on general practice workload [9].

The associated hospital activity for back pain was studied. We looked for variations in the data to see if there were issues that needed addressing, including by deprivation status (suggesting inequity) and variation by general practice (suggesting geographical or clinical unwarranted variation).

To overcome differences in the age-sex profile of patient groups, the results were standardised by applying category specific rates of each population to the European Standard Population (ESP, expressed per 10,000 population).

Because we are looking at limited time periods and relatively small subgroups of the population, binomial confidence intervals of 95% were applied to define the level of confidence we can have in the results. For some aspects of the analysis, to increase the sample size, we analysed the data over a multi-year time-period.

A list of all the major interventions we were providing for people with back pain was compiled, which included hospital and community-based interventions, such as physiotherapy, back pain classes, and pain management services. Some interventions were provided by multiple providers. We used locally held finance data to understand the number of people who received each service in 2019/20 and the cost associated with providing that service. We then used QALY assessments from a systematic review by the National Institute for Health and Care Excellence (NICE) of research studies that included an economic evaluation of back pain interventions- this review graded studies, outlining any possible limitations [10].

**Fig. 1:**
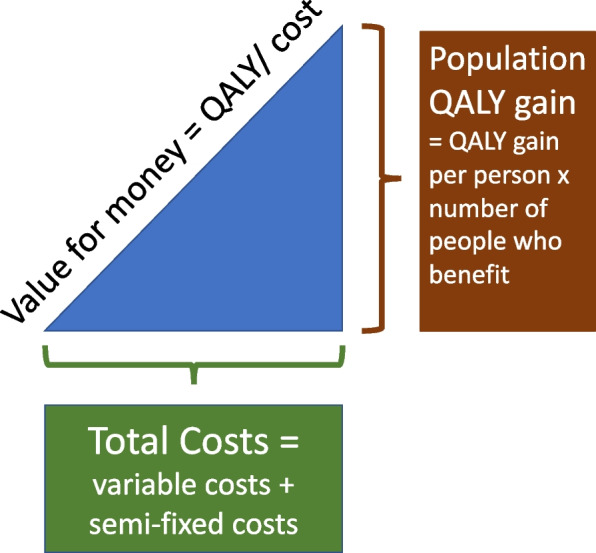
STAR value for money triangle

Using these data, we used the Socio-Technical Allocation of Resources (STAR) approach to examine and discuss the population benefit in relation to resource use and thereby reallocate resources to make value improvements [11]. STAR is designed for use by stakeholders to assess value for money of different interventions by using two visual models. The first visual model is the value for money triangle (see Fig. 1). Value is on the vertical axis, estimated by the QALY gain (number treated multiplied by mean gain in QALYs), costs are on the horizontal axis (fixed costs plus number treated multiplied by variable costs per patient), and value for money is as the hypotenuse of the triangle. The greater the value for money of an intervention, the steeper the slope of the hypotenuse. The second visual model is the efficiency frontier which stacks the value for money triangles in order of their slope – starting with the steepest (highest value for money). These are displayed in the results section below as Figs. 6 and 7.

## Results

**Fig. 2:**
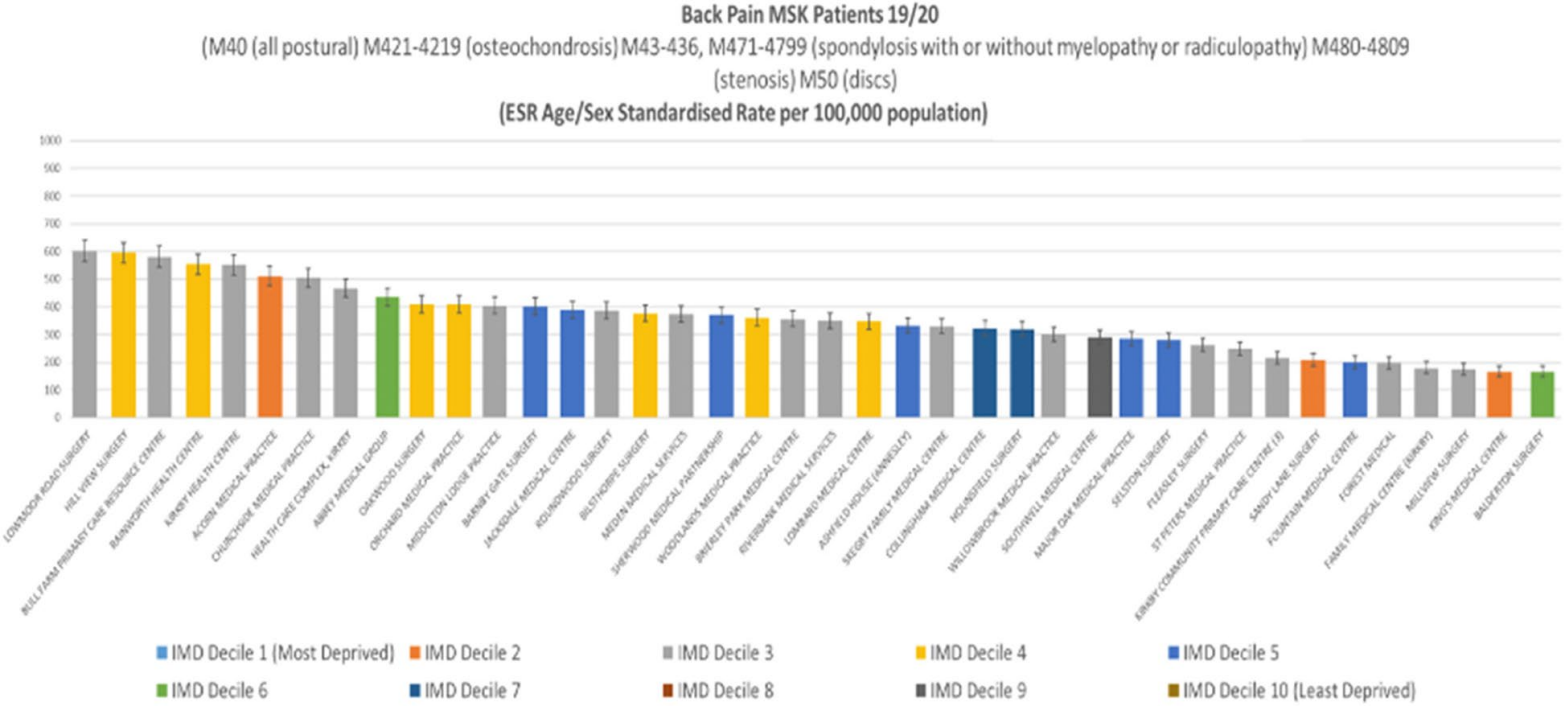
Mid-Nottinghamshire MSK secondary care patients with back pain 2019/20. Each bar represents a general practice

In 2019/20, there was considerable variation among GP practices in the numbers of patients treated in secondary care with a diagnosis of back pain after standardisation for age and sex. Deprivation, an indicator of workload in general practice, does not explain the variation (see Fig. 2).

**Fig. 3:**
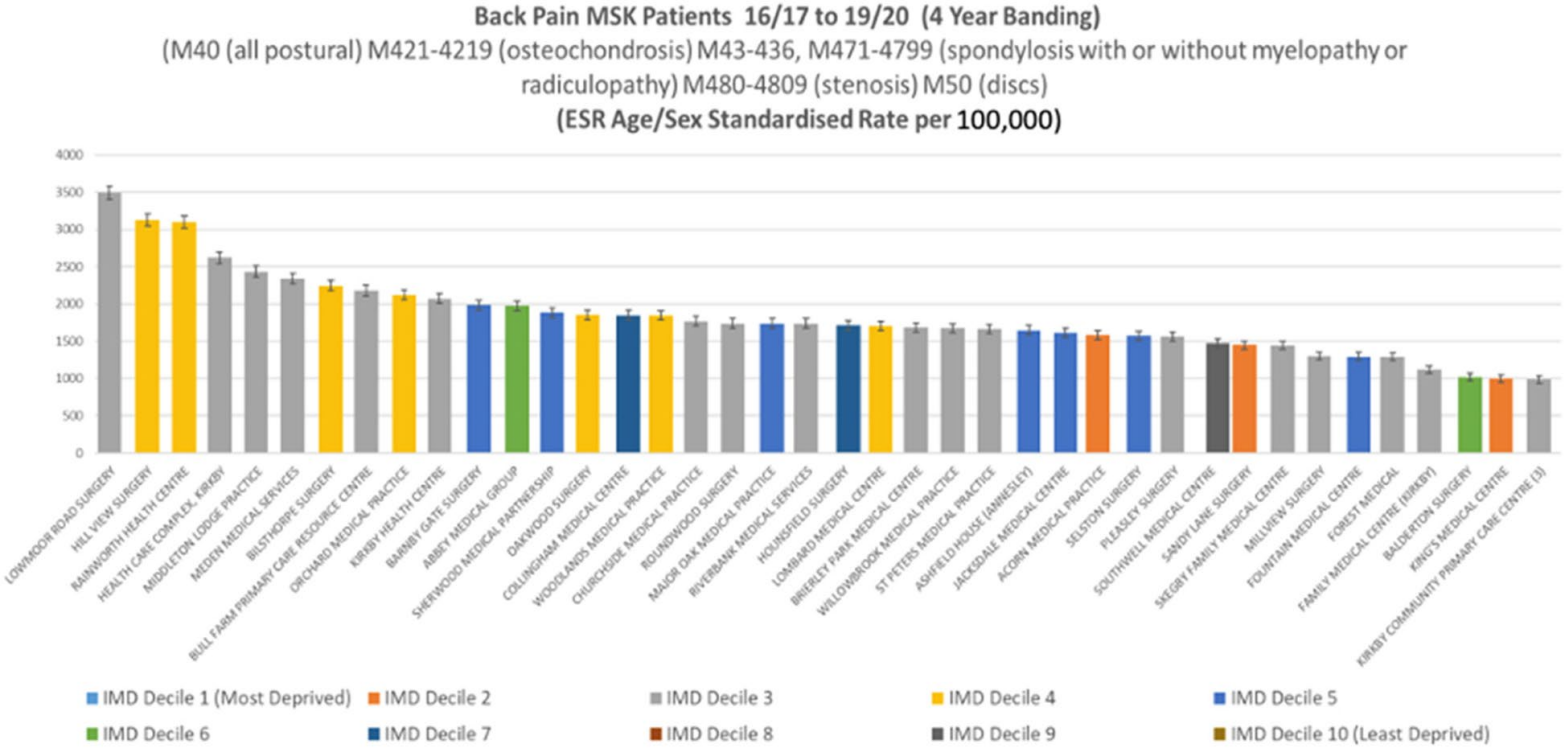
Mid-Nottinghamshire MSK secondary care patients with back pain 2016/17 to 2019/20. Each bar represents a general practice

Combining the data for the period 2016/17 to 2019/20, the variation widens (see Fig. 3).

**Fig.4:**
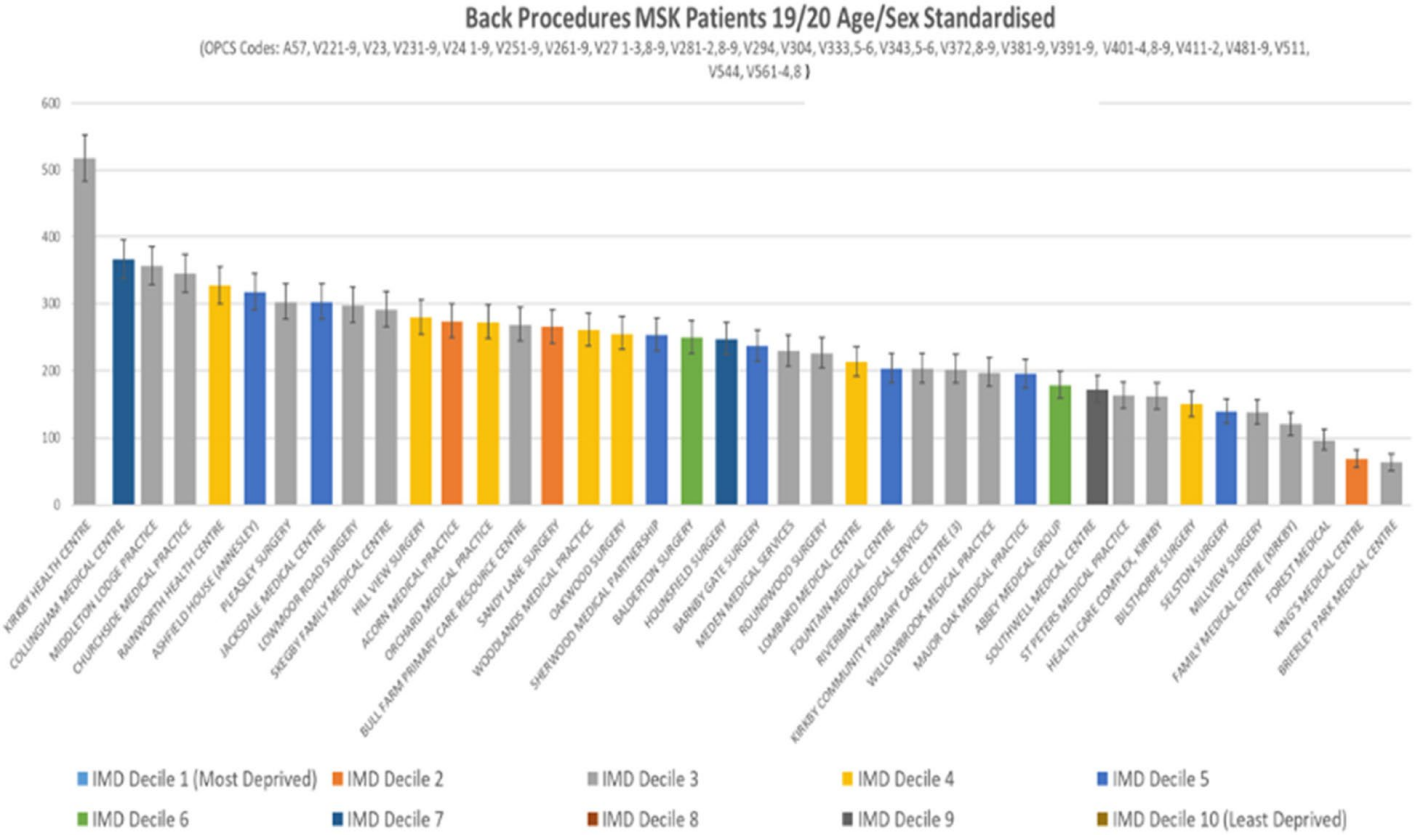
Back procedures for MSK patients, 2019/20 (age-sex standardised). Each bar represents a general practice

In 2019/20, there was almost an eight-fold variation among GP practices in the numbers of patients who had a back pain procedure (e.g., surgery or injection) in hospital (see Fig. 4). Again, deprivation does not explain the variation.

**Fig. 5:**
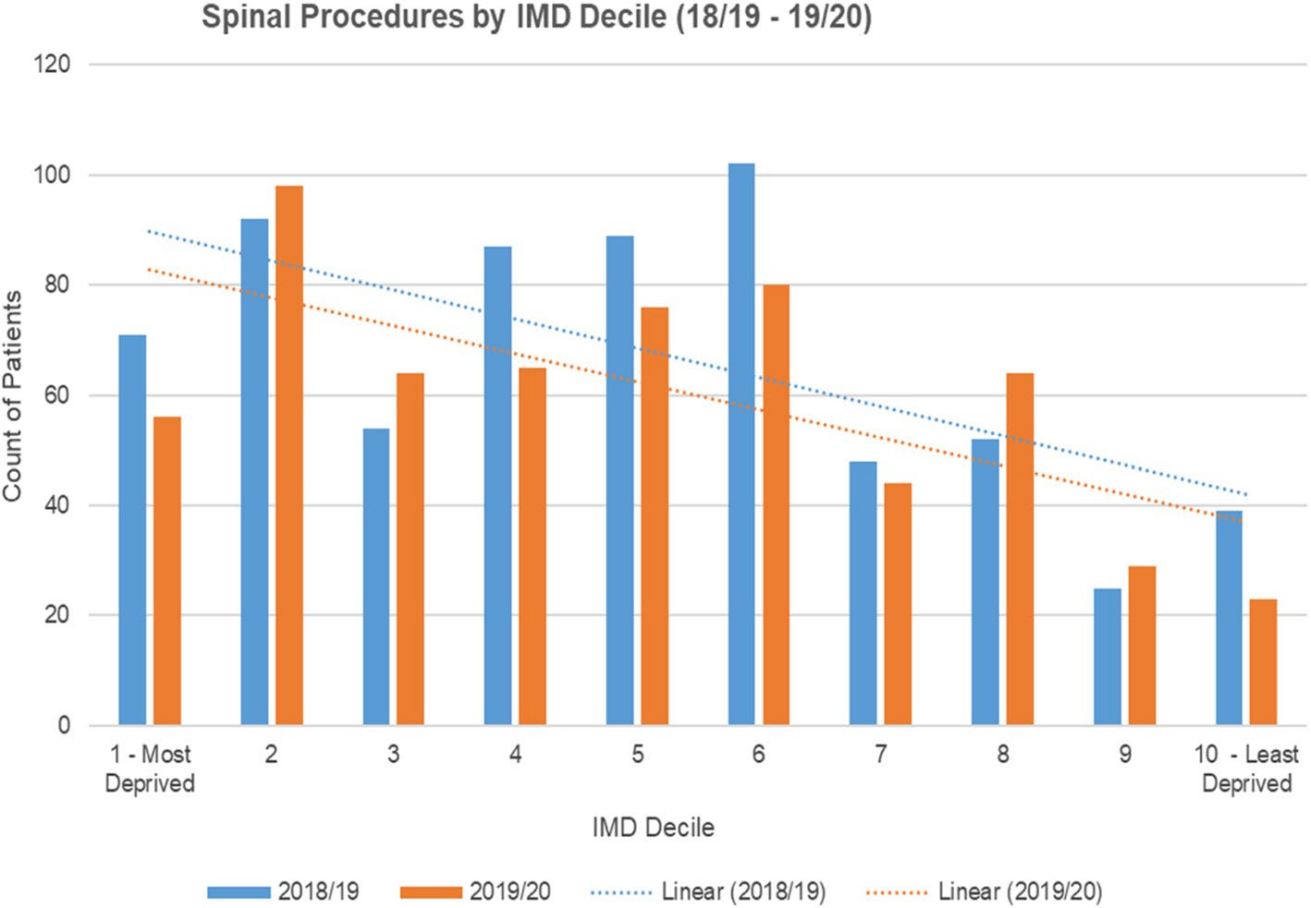
Spinal procedures by IMD decile 2018/19—2019/20

We found higher rates of back pain procedures when deprivation was measured by geographical area of patient residence. There appear to be lower spinal procedure rates among the least-deprived population groups (see Fig. 5, statistical significance unknown).

People in the most-deprived population groups have back procedures around 10–20 years younger than those in the least-deprived population groups, although the statistical significance of this is unknown (see Table 2).

**Table 2:**
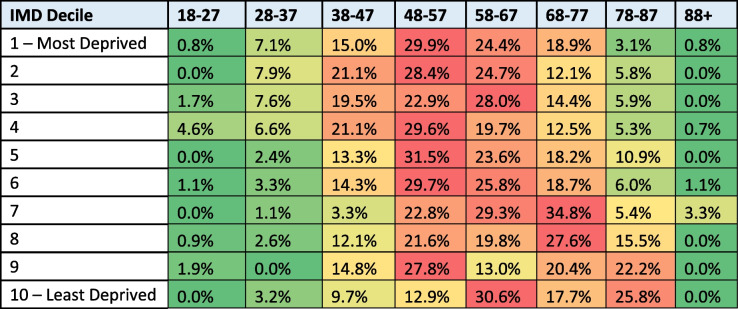
Percentage of people having back pain procedures by age and deprivation (key: red = highest; green + lowest)

In all, 29 separate interventions were being provided. We wanted to understand how many people were receiving each intervention, and how much resources was being consumed in doing so (note many people with back pain might have more than one intervention in the time period, either in parallel or serially). We therefore used health system records to establish the number of people receiving each intervention, the total expenditure on each intervention, and from the NICE systemtic economic review of low back pain, the research-derived quality adjusted life year (QALY) for each intervention, and the total number of QALYs provided by that intervention to the population receiving it (as estimated from the research evidence) is shown in Table 3.

**Table 3:**
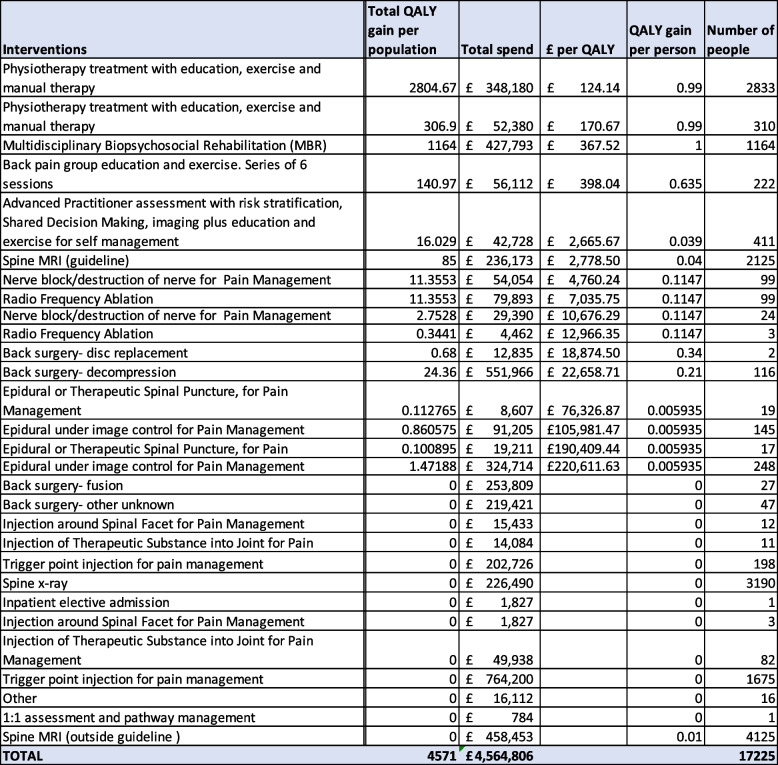
Interventions provided for back pain in Mid-Nottinghamshire, 2019-20 (Note that rows two and three are the same procedure offered by different providers. No further information was available on ‘in patient’ admissions or ‘other’)

For the interventions shown in Table 3 without a QALY, back surgery-fusion, X-ray spine, and several of the injections (namely, injection around spinal facet, injection of therapeutic substance into joint, trigger point injection), there were conclusive studies found in the NICE systematic review showing no benefit in quality of life for people with back pain [10]. MRI scans conducted outside of guidelines (calculated using a locally conducted audit of procedures) was likely to cause a worse quality of life in people with back pain. There was insufficient evidence for the other procedures to know whether they provided quality of life benefits. In all, 17,225 people received some form of treatment for back pain, associated with a spend of £4.5 million and an estimated QALY gain for the treated population of 4,571 QALYs.

**Fig. 6:**
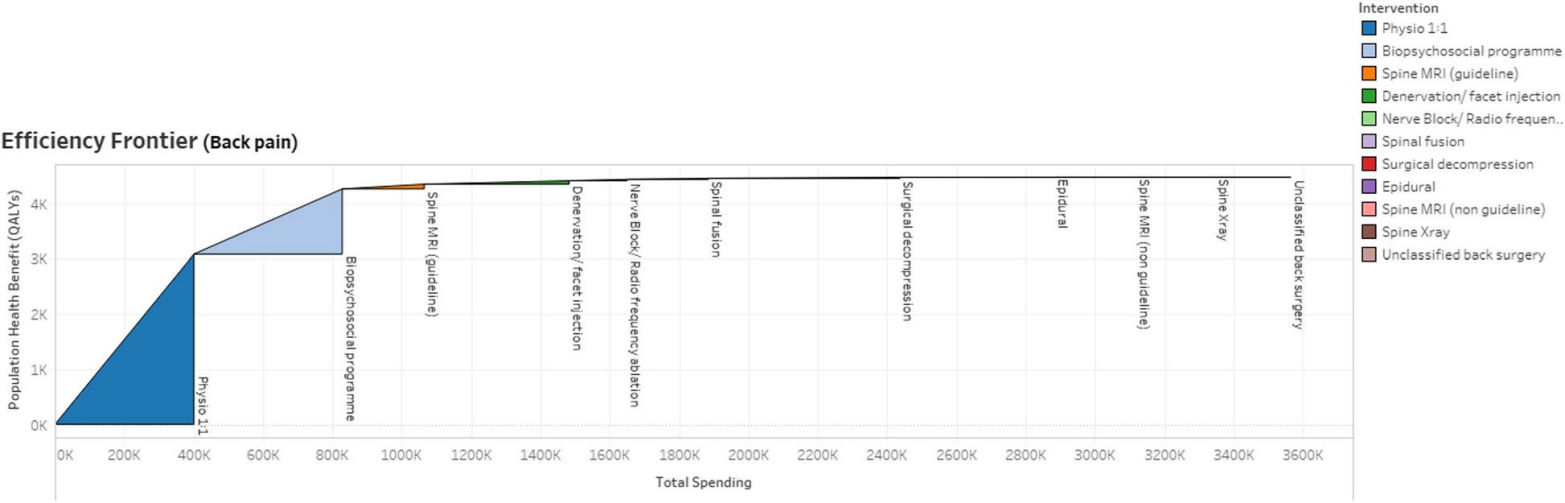
Visual representation of population benefit versus expenditure for back pain interventions in 2019/20 (simplified)

To focus efforts of reallocation where they would be most fruitful, we omitted interventions that were low in costs or volume, which reduced the total numbers of people in the population treated, and the total costs, but did not affect the total QALYs gained. We also consolidated other interventions into a single intervention, e.g., all injections of no benefit. The result is the frontier diagram shown in Fig. 6, which gave the MSK Together group an easy-to-understand visual representation of the core data shown in Table 3, and enabled them to discuss the relative merits of different interventions.

After discussions among the MSK Together group, it was agreed to reduce the number of MRI scans and spinal X-rays being done outside of guidelines and the number of ineffective injections. Estimates were made on the level of success of such efforts and the resources that could be released, noting that many fixed costs, such as those related to facilities, cannot be easily released for reinvestment.

The MSK Together group decided to reinvest those resources in digital support services for back pain (noting that they need to ensure that inequities in uptake/ provision are not exacerbated by such a service), community-based back pain classes (located preferentially in deprived areas) and case management for people with complex back pain. QALY estimates for these services were made by consensus discussions among the MSK Together group. Estimates of the costs of, and the number of people who would benefit from these interventions were made by a group of clinicians, managers, and finance experts (see Table 4). The MSK Together group, however, could not agree on what to do about spinal fusions, and this topic will be revisited in future rounds of value improvement activities.

**Table 4:** Estimated costs, QALYs and population treated for new interventions

Number of people treated	Cost per person	QALY per person	Cost per QALY	Total invested	Total QALY gain
10,000	£2	0.07	£29	£20,000	700
3,000	£50	0.67	£75	£150,000	2,010
200	£500	1.90	£263	£100,000	380
			**TOTAL**	**£270,000**	**3,090**

**Fig. 7:**
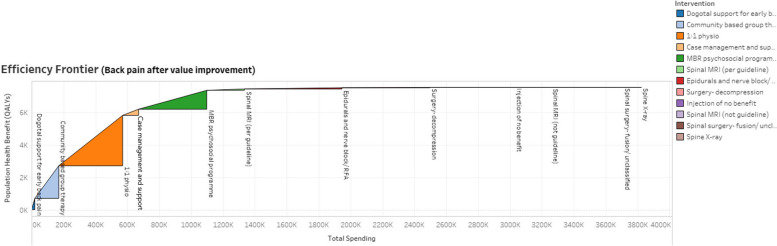
Visual representation of population benefit versus expenditure for back pain interventions after value improvement discussions (simplified)

Figure 7 gives the efficiency frontier diagram after the reallocation of resources. We estimated that, for the population of over 25,000 people treated for back pain within the same total spend of £4.5 million, the potential total gain would be 7,661 QALYs (a 67% increase) and 10,000 additional people with access to digital support services (representing a 46% increase in the number of people receiving an intervention).

## Discussion

This project was designed to allow a group of people working collectively in MSK care (patients, clinicians, and managers) in Mid-Nottinghamshire, England, to use pragmatic approaches to analyse variation and study the cost–benefit of interventions to understand where, for people with back pain, they could reallocate resources to deliver on the NHS triple aim, improving value, increasing equity, and enhancing the sustainable and efficient use of resources [1].

Analyses of variation and efficiency frontier diagrams showed that considerable amounts of resources were being used on low or lower value interventions. The way resources are used in the English NHS’s systems of care have evolved over time and were never designed to be optimal. That is why projects such as that in Mid-Nottinghamshire continue to discover a suboptimal use of resources [12]. Because this is a novel approach, it was difficult to collect the necessary data. The data collected routinely by health services are designed to account for activity and finance by institutions, not according to the benefits of interventions, nor the needs of populations. The reintroduction of national programme budgeting comparison tools to the NHS would help generate the data required [13].

Although some practitioners see technical obstacles to the approach we have taken, particularly regarding the estimation of QALYs, these are easily overcome and any uncertainty over QALY values can be explored through group discussion. What was vital, however, was the development of the MSK Together group who were willing to work collectively to deliver on the NHS Triple Aim and act as stewards of the common pool resources available for the treatment of back pain. This project was made possible only through the development of a culture of stewardship among the MSK Together group. Insights from this mutli-disciplinary group, including those of the patients, were critical to making reasonable decisions that took account of local context. For instance, general practitioners (GPs) in the UK have faced large increases in demand, while the size of the workforce has stayed the same. We learnt that in a busy clinic, it is easier for GPs to refer back pain patients for an MRI, than to provide advice and education.

Work is already underway to implement the decisions made by the MSK Together group regarding disinvesting and reinvesting resources to improve the level of benefit for people with back pain in Mid-Nottinghamshire without the need for extra resources.

A co-produced website is in publication, displaying trusted advice and links to education, exercise, and information about back pain as well as other community services to empower people to self-manage their condition holistically. With readily available paper and video versions of the information to reduce digital exclusion, this locally produced resource is offered to patients who have early back pain or are at low risk of chronicity at initial presentation in general practice.

MSK Together are engaged with and supporting GP and MSK First Contact Practitioners (FCP- physiotherapists) to risk-stratify care and facilitate direct access to higher value investments with wholescale GP and FCP training delivered by the members of the MSK Together group. The gym-based exercise and education classes are being delivered in more-deprived areas and at no cost to the patient. The virtual multi-disciplinary team (MDT) is directly accessible via the FCPs in general practice to discuss and co-ordinate care and support shared decision-making conversations for people with more complex back pain or where surgery might be helpful. Bearing in mind the pressures on general practice described above, this is an easy, and high value, route for a busy GP to direct a patient. The MSK Together group anticipates a reduction in unwarranted variation, improved personal and population outcomes, and a reduction in MR images that do not improve outcomes and injections that have no evidence of benefit, thereby freeing up further resources for reallocation.

Despite the challenges in gathering data, specifically outcome data, and aggregating datasets to enable a population view, over the longer term, MSK Together group plans to monitor outcomes, activity and resources, as well as access to services, which will provide detailed intelligence to enable work with specific cohorts in the future, making continuous value improvement within a culture of stewardship the norm.

The final point we make is this. There is an ocean of studies documenting the endemic nature and scale of unwarranted variations in medical care. By comparison, the number of studies showing how unwarranted variation can be reduced, other than through shared decision making, are a drop in that ocean. We see the creation of a group like MSK Together, using the STAR approach, as a way of reducing unwarranted variations by focusing efforts on the reallocation of resources from lower value to higher value interventions.

### Limitations

We did not look at the use of painkillers, which form an important part of the interventions used in back pain. This is chiefly because it would be impossible to find which people were taking painkillers specifically for back pain and would have crated considerably extra work. There are prescribing programmes underway in the NHS to rationalise the use of painkillers, including for conditions such as back pain.

The success of elements of this work were highly dependent on the actions taken to create the conditions in which the MSK Together group could work collectively, notably creating the right cultural conditions and training the group in the underlying principles of value improvement. This is not easily reproducible, but the necessary skills could probably be taught.

This was a pragmatic piece of work and did not constitute a ‘research study’ nor did it involve extensive note reviews (other than the audit of MRI scans). To that extent we may have missed other opportunities for improvement by excluding emergency and trauma care, for instance, which could be addressed at a later date, when we have exhausted all the improvement opportunities we did find.

### Implications for practice

Using the results of an analysis of variation can allow healthcare providers to improve the value of healthcare, giving insights into where to focus efforts, especially when paired with local knowledge and understanding. It would be tempting to spend extensive time risk adjusting the data, or conducting extra studies, but we did not find this necessary and would have added to the time needed and resources required to conduct the work; perfection is the enemy of the good. We have found that looking at variation in access to, and possibly inequities, high-quality healthcare is a strong motivator for change among different stakeholders. Further, looking at where resources are used and linking these with available high quality systematic economic reviews (NICE in this instance) has allowed us to find ways of using resources better.

## Data Availability

The sources of the data and material is described in the paper. It is a mix of publicly available data and pseudonymised patient level data managed by NHS England.
